# Voltammetric determination of phenylephrine hydrochloride using a multi-walled carbon nanotube-modified carbon paste electrode

**DOI:** 10.1098/rsos.181264

**Published:** 2018-12-05

**Authors:** Q. Zhou, H. Y. Zhai, Y. F. Pan

**Affiliations:** 1 Department of Pharmacy, GuangDong Pharmaceutical University, Guangzhou 510006, People's Republic of China; 2 Department of Chemistry, GuangDong Pharmaceutical University, ZhongShan 528458, People's Republic of China

**Keywords:** voltammetric determination, phenylephrine hydrochloride, multi-walled carbon nanotube, modified carbon paste electrode

## Abstract

A chemically modified carbon paste electrode (CPE) was designed by mixing graphite and multi-walled carbon nanotubes (MWCNT). The electrochemical behaviour was studied, and the determination method of phenylephrine hydrochloride (PHE) on this sensor was established. According to the results, the optimal ratio of MWCNTs was approximately 12.5% (w/w). MWCNT-modified carbon paste electrodes (MWCNT-CPEs) showed high electrochemical activity for PHE, producing a sharp oxidation peak current (*I*_p_) at approximately +0.816 V versus a saturated calomel electrode (SCE) reference electrode in phosphate buffer solution (PBS, pH 6.45), and the *I*_p_ increased by approximately two times compared to that of the bare CPE. The anodic *I*_p_ was linearly related with 5.0 × 10^−6^–7.5 × 10^−4^ mol l^−1^ PHE, with a detection limit of 3.7 × 10^−7^ mol l^−1^. Furthermore, MWCNT-CPEs were successfully applied to the determination of PHE in injection, eye drop and nasal spray liquid samples as a simple, rapid and low-cost method.

## 1. Introduction

**Scheme 1.**
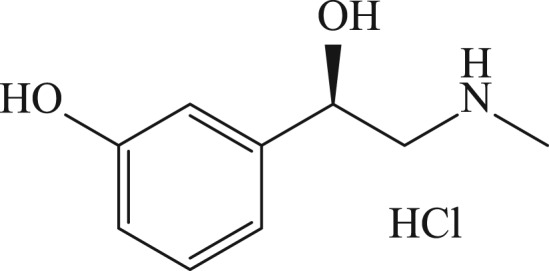
Chemical structure of PHE.

Phenylephrine hydrochloride (PHE) (scheme 1) is an adrenergic receptor agonist that acts on *α* receptors in skin, mucosa, viscera and other tissues, to contract blood vessel and increase blood pressure. Clinically, it is mainly used to maintain a stable blood pressure during shock and under anaesthesia, and it can also be used to eliminate inflammation of nasal mucosa, dilate the pupils, etc. Pharmaceutical dosage forms are mainly injection, eye drops and nasal spray liquid and concentrations are within 0.1–1% (w/v).

At present, determination methods of PHE include HPLC [1,2], UPLC-MS [3], spectrophotometry [4,5] and capillary electrophoresis [6,7]. The pretreatment processes of these methods are relatively complex, requiring large-scale instruments. Thus, they are not convenient for on-site quick detection. The sample pretreatment process of electrochemical determination is relatively simple, i.e. it is a convenient and fast measuring process using inexpensive instruments. Meanwhile, a chemically modified carbon paste electrode (CM-CPE) can improve selectivity and sensitivity of the CPE and integrate separation, enrichment and selective determination into a single step; therefore, it is one research focus of the analysis and detection field [8–10]. Our research group has made some achievements in the research of modified CPE [11–13].

It has also been reported that a modified glassy carbon electrode (GCE) can be used to detect PHE [14,15], while reports on using modified CPE to detect PHE are relatively rare [16,17]. Because of the high sensitivity and convenience in making and updating multi-walled carbon nanotube carbon paste electrodes (MWCNT-CPEs), this paper used MWCNT-CPEs as the working electrode, researched the electrochemical behaviour of PHE on the electrode and established the method of direct determination of PHE. Furthermore, different dosage forms were detected, such as injection, eye drops and nasal spray liquid, with satisfactory results. The method does not require complex pretreatment of samples and combined with a small portable electrochemical workstation, samples can be detected on site, i.e. there is no need to send them to professional laboratories.

## 2. Experimental set-up

### 2.1. Apparatus and chemicals

Cyclic voltammetry (CV) was performed on an Ingsens-1010 series hand-held electrochemical workstation (Ingsens Instruments, Guangzhou, China). A conventional three-electrode system with an MWCNT-CPE (home-made) as the workstation, a saturated calomel electrode (SCE; Chenhua Instruments, Shanghai, China) as the reference electrode and a platinum electrode (Chenhua Instruments, Shanghai, China) as the indicating electrode was used. A pH meter (pHS-25, Leici Instrument, Shanghai, China) with a double junction glass electrode was used to check the pH of the solutions.

PHE (99%), MWCNTs (SP, 3–5 nm in diameter), graphite powder (SP, 40 nm) and liquid paraffin (HPLC) were purchased from Shanghai Aladdin Reagent Co., Ltd. Other chemicals were analytically pure, and the water used in the experiment was deionized water.

### 2.2. Preparation of solutions

A 1.0 × 10^−3^ mol l^−1^ PHE solution was prepared by dissolving 20.37 mg of PHE standard substance in 100 ml of deionized water, and the solution was kept in a refrigerator at 4°C. Phosphate buffer solution (PBS) with different pH values was prepared by mixing stock solutions of NaH_2_PO_4_ and KH_2_PO_4_, and the pH was adjusted with H_3_PO_4_ and NaOH.

The product instruction manual showed that the main component of PHE injection is deoxidation PHE, and the auxiliary materials are sodium metabisulfite, sodium chloride and edetate disodium. The main components of compound tropicamide eye drops are tropicamide and PHE. The main components of compound PHE nasal spray liquid are PHE, dexamethasone sodium phosphate and lincomycin hydrochloride. The above samples did not need pretreatment. An appropriate amount of sample can be directly measured and was diluted with PBS (pH = 6.45) to the required concentration.

### 2.3. Preparation of MWCNT-CPE

MWCNT-CPE was made by mixing graphite powder, MWCNTs and liquid paraffin evenly with a certain ratio. The mixture was placed in a glass tube with a radius of 2.0 mm and compacted. Copper wire was used as a guide line, and the electrode was fixed after drawing off the guide line. Then, the surface of the electrode was smoothed and polished on a piece of smooth white paper.

Based on the above methods, we also obtained bare CPE with no MWCNTs added.

### 2.4. Electrochemical analysis procedure

An appropriate amount of PHE solution was placed in a small beaker, and the three-electrode system was inserted. The enrichment time was adjusted to 10 s and the scan speed was 400 mV s^−1^. The initial and final potentials were from 0.2 to 1.0 V, and the peak voltage (*E*_p_), peak current (*I*_p_) and other information were recorded.

After the detection was completed, the working electrode was placed into a 0.1 mol l^−1^ NaOH solution and cyclically scanned 10 times to clear PHE adsorbed on the electrode.

## 3. Results and discussion

### 3.1. Characteristics of the MWCNT-CPE using cyclic voltammetry

**Figure 1.**
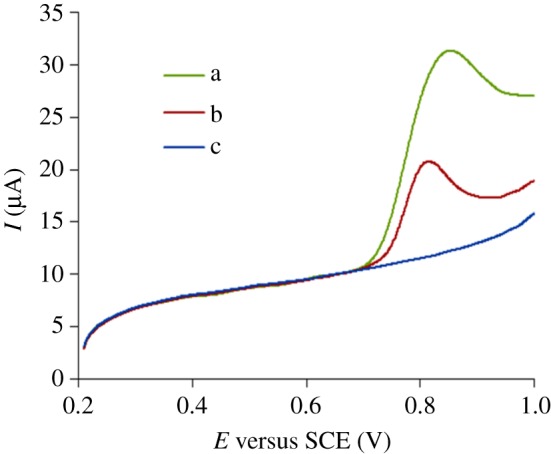
Cyclic voltammograms of 5.0 × 10^−5^ mol l^−1^ PHE for MWCNT-CPEs (a) and bare CPE (b) in phosphate mixture (pH 6.45), and bare CPE in blank phosphate mixture (c) at a scan rate of 400 mV s^−1^.

The electrochemical behaviour of PHE was researched using MWCNT-CPE and CPE, i.e. two different kinds of electrodes, to, respectively, scan in blank or 5.0 × 10^−5^ mol l^−1^ PHE solution (figure 1). The anodic *E*_p_ is approximately 0.82 V, and no cathode peak is observed, which shows that PHE oxidation is irreversible on the electrode. The *I*_p_ of PHE on CPE (b) is relatively less; and the *I*_p_ is increased by approximately two times on MWCNT-CPE (a).

### 3.2. Effect of amount of MWCNTs

**Figure 2.**
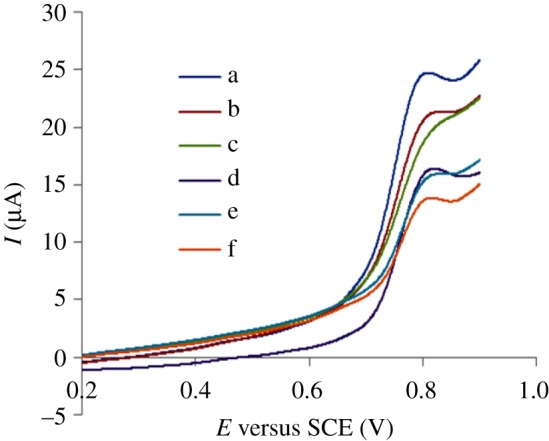
Effect of MWCNTs amount on the *I*_p_ of 5.0 × 10^−5^ mol l^−1^ PHE: (a) 12.5%, (b) 11.1%, (c) 10.0%, (d) bare CPE, (e) 14.3%, (f) 16.7%.

To obtain the optimal conditions to improve electrochemical performance, the ratio of MWCNTs added was studied. MWCNTs were mixed with graphite powder with a certain ratio, and an appropriate amount of liquid paraffin was added to obtain five MWCNT-CPEs. Scanning was conducted in 5.0 × 10^−5^ mol l^−1^ PHE solution (figure 2), and it shows that when the ratio of MWCNTs and graphite powder is 12.5% (figure 2*a*), the *I*_p_ is relatively higher, and the peak shape is better.

### 3.3. Effect of buffer solution

Britton–Robinson (BR), KH_2_PO_4_-H_3_PO_4_, Na_2_HPO_4_-citric acid, NaAc-HAc, NH_3_-NH_4_Cl, PBS and other buffer solutions were used to dilute PHE solutions to 5.0 × 10^−5^ mol l^−1^, and cyclic scanning was conducted on the above solutions. We found anodic peaks for BR, KH_2_PO_4_-H_3_PO_4_, NH_3_-NH_4_Cl and PBS, while the peak shape in BR is not obvious and is unstable, and the *E*_p_ is approximately 0.60 V; *E*_p_s in NH_3_-NH_4_Cl and PBS are approximately 0.82 V. The peak shape in PBS is better, and the *I*_p_ is higher. Furthermore, various experiments show that reproducibility in PBS is also better.

### 3.4. Effect of pH

**Figure 3.**
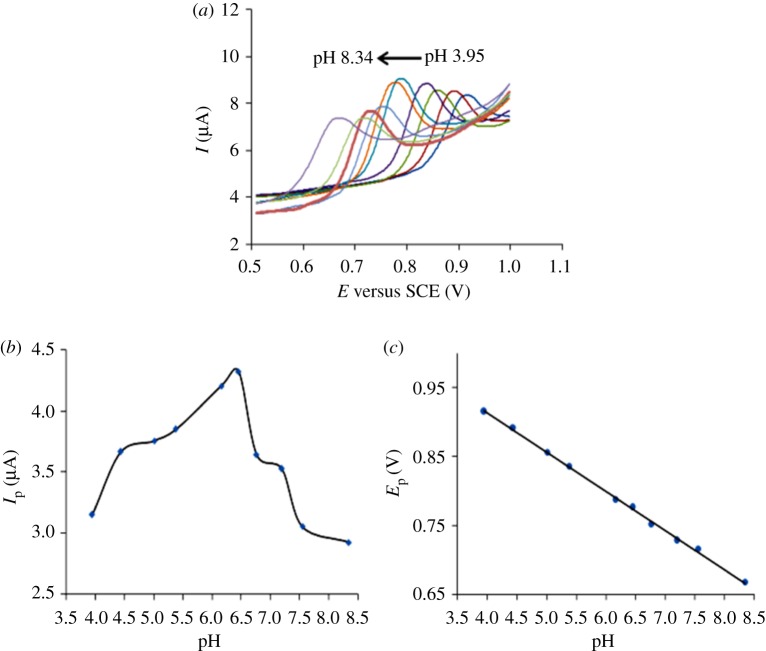
(*a*) Cyclic voltammograms of 5.0 × 10^−5^ mol l^−1^ PHE in phosphate mixture at pH 3.95–8.34 from right to left. The effect of pH on the peak (*b*) current and (*c*) potential.

The influence of pH on *I*_p_ (5.0 × 10^−5^ mol l^−1^ PHE) by CV in PBS with various degrees of acidity was examined, and according to figure 3, the *I*_p_ increases to its peak and then decreases (figure 3*b*). When the pH is 6.16 and 6.45, the *I*_p_ is larger and the peak pattern is better (figure 3*a*); therefore, PBS at pH of 6.45 is chosen in the following experiment.

Furthermore, it is also found that PHE *E*_p_ decreases with the increase in pH, and there is also a linear relationship between them (figure 3*c*), which indicates that protons are involved in the PHE oxidation reaction. When the pH is within 3.95–8.34, the linear equation between *E* and pH is *E* = 1.1468–0.0571 pH (*r* = 0.9991), and the slope is 0.057. According to the Nernst equation, the proportion of electrons to protons participating in the redox reaction is 1 : 1 based on the slope of 0.059. Thus, PHE on MWCNT-CPEs is a redox reaction with an electron-to-proton ratio of 1 : 1.

### 3.5. Effect of accumulation time

The influence of accumulation time on the *I*_p_ for two different PHE concentrations was investigated, and when the concentration is 1.0 × 10^−4^ mol l^−1^, the *I*_p_ reaches the maximum in 10 s. When the concentration is 5.0×10^−5^ mol l^−1^, with increasing accumulation time, there is no significant change in *I*_p_; therefore, the accumulation time in the following experiment is 10 s.

### 3.6. Effect of scan rate

**Figure 4.**
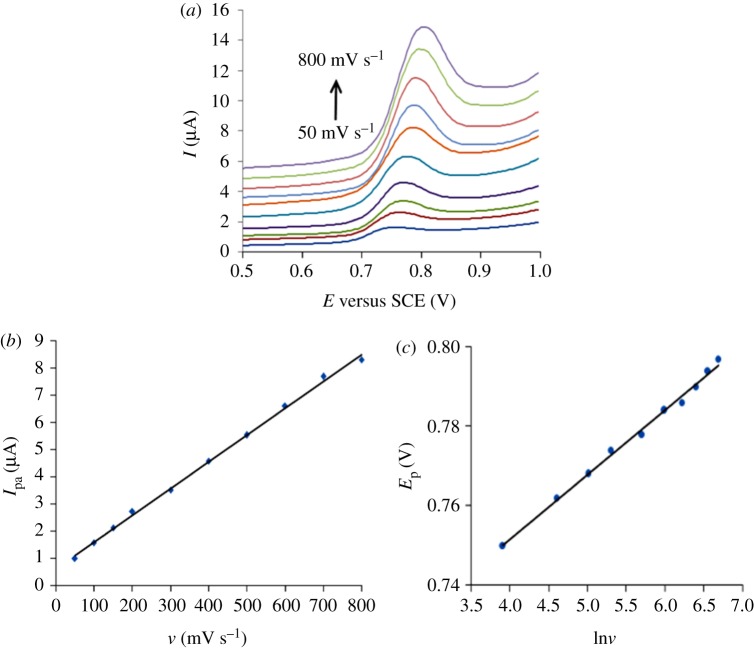
(*a*) Cyclic voltammograms of 5.0 × 10^−5^ mol l^−1^ PHE in phosphate mixture at different scan rates of 50–800 mV s^−1^. (*b*) Linear relationship of *I*_p_ versus scan rate. (*c*) Linear relationship of *E*_p_ versus ln*v*.

The effect of scan rate on the electrochemical behaviour of PHE was investigated at the MWCNT-CPE by CV. In PBS-buffered solution with pH 6.45, accumulation time of 10 s, and PHE concentration of 5.0×10^−5^ mol l^−1^, the scan rate was changed from 50 to 800 mV S^−1^, and the changes in *I*_p_ and *E*_p_ of PHE were recorded (figure 4*a*); with an increasing scan rate, the *I*_p_, as well as *E*_p_, gradually increases.

In the experiment, it is also found that there is a good linear relationship (figure 4*b*) between the *I*_p_ and scan rate. The linear equation of *I* and *v* is *I* = 0.0099*v* + 0.6147 (*r* = 0.9990), which illustrates that the redox reaction of PHE on MWCNT-CPE is mainly adsorption controlled.

In addition, there is also a good linear relationship (figure 4*c*) between *E* and the ln*v*, and the linear equation is:

3.1 E=0.0223lnv+0.6863 (r=0.9974).

According to reversible electrode reactions, the following relationship exists between the *E*_p_ and scan rate [18]:

3.2 $$E_{p}=E^{\theta }-\frac{RT}{\alpha nF}\left[0.78-\ln\frac{k^{0}}{D^{1/2}}+\ln{\left(\frac{\alpha nFv}{RT}\right)}^{1/2}\right],$$

where *α* is the charge transfer coefficient, *n* is the number of transferred electrons, *R* is the gas constant (8.314 J K^−1^), *T* is the temperature (K, 298) and *F* is the Faraday constant (96487 C mol^−1^).

**Scheme 2.**
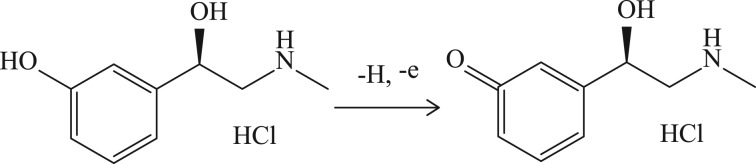
Oxidation mechanism of PHE at MWCNT-CPE.

Combining equation (3.1) with (3.2), 1/2 × *RT*/*αnF* = 0.0223, and *αn* is calculated to be 0.58. The value of *α* is assumed to be 0.5 for an irreversible electrochemical process [19], and the electron participating in the reaction is calculated as 1.16 (close to 1). Combining the conclusions from before, the reaction mechanism of PHE on MWCT-CPE is shown in scheme 2.

### 3.7. Interference studies

Some common substances or constituents of pharmaceutical formulations were added to a 5 × 10^−5^ mol l^−1^ PHE solution, and the changes in *I*_p_ and *E*_p_ were measured (table 1). The result shows that these substances have no interference on PHE detection, and this sensor is highly selective for PHE.

**Table 1.** Effect of various substances on the *I*_p_ and *E*_p_ of PHE.

substances	amount (mol l^−1^)	*I*_p_ (µA)	relative error (%)	*E*_p_ (V)	relative error (%)
none		10.99		0.816	
NaCl	0.05	11.11	1.82	0.820	0.49
CaCl_2_	0.05	10.41	−5.28	0.817	0.12
KNO_3_	0.05	10.87	−1.09	0.816	0
BaCl_2_	0.05	10.42	−5.29	0.819	0.37
Na_2_SO_3_	0.005	10.89	−0.91	0.816	0
Na_2_-EDTA	0.005	10.92	−0.64	0.816	0
tropicamide	0.005	11.22	2.09	0.823	0.86
dexamethasone sodium phosphate	0.005	10.97	−0.18	0.815	–0.12
lincomycin hydrochloride	0.005	11.11	1.82	0.820	0.49

### 3.8. Electrode precision and repeatability

After obtaining a stable scan in the blank solution, the freshly prepared modified electrode was evaluated by continuously measuring the PHE standard solution 10 times. As the relative standard deviation (RSD) was 0.7%, the modified electrode was highly precise. Seven electrodes were prepared with the same condition at the same time. RSD was 2.9% when measuring the same PHE standard solution, which indicated good repeatability.

### 3.9. Calibration curve and limit of detection

**Figure 5.**
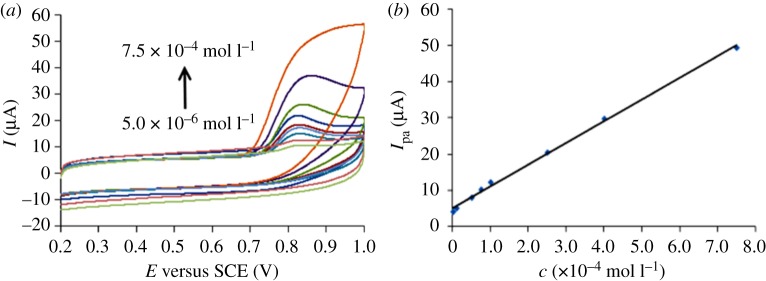
(*a*) Cyclic voltammograms of PHE in phosphate mixture at different concentrations of 5.0 × 10^−6^–7.5 × 10^−4^ mol l^−1^. (*b*) Linear relationship of *I*_p_ versus PHE concentration.

Under the optimized conditions, CV was selected to determine PHE at MWCNT-CPEs. When the PHE concentration is within 5.0 × 10^−6^–7.5 × 10^−4^ mol l^−1^, there is a good linear relationship (figure 5) between PHE concentration and *I*_p_. The linear equation is *I* = 5.8764*c* (×10^−4^mol l^−1^) + 5.737 (*r* = 0.9995), and based on the signal-to-noise ratio of 3, the detection limit is obtained as 3.7 × 10^−7^ mol l^−1^.

### 3.10. Sample analysis

MWCNT-CPE was used to measure PHE injection, eye drops and nasal spray liquid. Referring to table 2 for the measurement results and recovery, it shows that the method is applicable for the determination of PHE in various medicines.

**Table 2.** Application of MWCNT-CPE for the detection of PHE in real samples.

samples	found (×10^−4^ mol l^−1^)	added (×10^−4^ mol l^−1^)	determined (×10^−4^ Mol/l)	recovery, *n* = 5 (%)	RSD, *n* = 5 (%)
injection	5.04	4.00	9.09	101.2	1.1
	5.04	5.00	10.00	99.20	0.7
	5.04	6.00	11.14	102.0	0.7
eye drops	1.32	1.00	2.27	95.00	3.5
	1.32	1.30	2.66	103.2	1.7
	1.32	1.60	2.89	98.13	2.5
nasal spray	0.62	0.40	1.05	107.5	2.2
	0.62	0.60	1.23	101.7	3.2
	0.62	0.80	1.42	100.0	4.1

## 4. Conclusion

A sensitive method of determining PHE with a selective MWCNT-CPE was established. The optimal conditions were as follows: the ratio of MWCNTs was approximately 12.5% (w/w) in the working electrode, PBS at pH 6.45 was chosen as the buffer and the accumulation time was 10 s. With a scan rate of 400 mV s^−1^, PHE exhibited an oxidation peak at 0.816 V. When the concentrations were 5.0 × 10^−6^–7.5 × 10^−4^ mol l^−1^, there was a good linear relationship between PHE concentration and *I*_p_, and the detection limit was found to be 3.7 × 10^−7^ mol l^−1^ (*S*/*N* = 3). The method was applied to detect PHE in different samples, and the results were satisfying. This method features simplicity, speediness and low detection cost using a small, portable electrochemical workstation, and it can be applied for quick on-site detection.

## Data accessibility

All data are included in the article.
